# Analysis of latent profiles and affected factors according to the implementation of a protection system against customer bullying in Korea

**DOI:** 10.1186/s12889-022-12743-7

**Published:** 2022-02-19

**Authors:** Eun-Mi Baek, Eun-Hi Choi, Hye-Sun Jung

**Affiliations:** 1 Department of Preventive Medicine, College of Medicine, Catholic University of Korea, Seoul, Republic of Korea; 2 Department of Nursing, Eulji University, 712, Dongil-ro, Uijeongbu-si, Gyeonggi-do, 11759 Republic of Korea

**Keywords:** Customer service, Bullying, Protection system, Latent profile types

## Abstract

**Background:**

Instances of customers bullying employees at the workplace are increasing with the development of the service industry. Korea has established a worker protection system to prevent negative effects of customer bullying on workers’ health. This study identified the latent profile types of protection against customer bullying in workplaces, and determined their predictors.

**Methods:**

Data were collected over 28 days, from March 2 to 30, 2020. This study identified whether protection against customer bullying is implemented for workers in person-to-person services, the change effected by this protection, and worker monitoring scope. Data from 1,537 out of 1,550 participants were analyzed, excluding the missing values. Latent profile types were identified using Mplus 8.5 for data analysis, and the multinomial logistic regression analysis was performed to review the predictors.

**Results:**

The results are as follows. There were four types of latent profile types: lagging, medium, relative preventive type, and excellent type. Variables predicting these types included age, gender, service period, position, occupational category, worker monitoring scope, decrease in the number of customers causing problems, decrease in disputes with customers, and worker satisfaction.

**Conclusions:**

First, a system to prevent customer bullying must be adopted in the service industry. Second, there must be a way to improve trust between customers and workers when protective measures are being adopted. Third, managers must establish a system that can both protect workers against bullying and provide customer satisfaction.

## Background

Korea’s industries are gravitating toward its booming service industry, which is experiencing a surge in workforce. According to Statistics Korea, 2.98 million business units are operating in the service industry nationwide, with 12.53 million workers as of December 2019—a 26% increase from 2.36 million business units and 42.2% increase from 8.81 million workers in 2009 [1].

The country’s service industry professes a customer-centric culture in which the customer is always considered correct—this, incidentally, is also the main reason for customers bullying workers. Bullying refers to being exposed to continuous and long-term mistreatment or abuse in a situation where one has difficulty defending himself or herself [2]. It is also a repeated negative behavior of disregarding an individual or a group, making scornful remarks, and spreading vicious rumors [3, 4]. Countless employees across multiple sectors experience workplace bullying on a regular basis [5]. However, in the service industry, there are increasing number of cases of third-party bullying of employees—that is, at the hands of customers. In Europe, 57.4% of service industry workers experienced bullying and violence by a third party in 2015 [6].

Given that the buying behavior of customers leads to profits for business owners [7], it is not easy for businesses to control customer bullying, even when it causes workers to experience deterioration of physical health (musculoskeletal pain, tension, headache, fatigue, etc.) and mental health (depression, anxiety, fear, etc.), thereby exerting a negative effect on their quality of life [8, 9]. It may also lead to negative results for organizational performance, such as decrease in job satisfaction, reduction of workplace involvement, and increase in turnover intention [10]. Previous studies have argued for workplace safety programs to prevent these negative effects on workers [11], and for effective bullying mitigation measures and employee training within the workplace [12].

Europe has already established national-level policies for worker protection against third party and for prevention and resolution of violent acts and bullying; it has begun showing effect [13]. The United States also has worker protection systems in each state. For example, Washington state pays subsidies for safety equipment, facility improvement, and educational programs under the Protecting Social Workers and Health Professionals from Workplace Violence Act of 2019 [14]. Canada requires companies to protect their employees according to the industrial health and safety standards and Part II of the Canada Labor Code, and all relevant information is updated monthly for smooth communication and settlement [15].

Korea also established new mandatory clauses for business owners regarding worker protection against customer bullying under the Occupational Safety and Health Act in October 2018 to prevent customer bullying and its negative impact on health of workers. The new regulations include posting texts or providing voice announcements to prevent abusive language use and health impairments due to customer violence, creating a customer service manual and providing education, suspending operations and transferring workers who experienced violence, prohibiting unfair treatment of workers, increasing rest time, offering therapy and counseling, and providing support necessary for damage claims [16].

The Occupational Safety and Health Act was specifically introduced for workers’ health protection. It has the objectives of preventing customer harassment, providing, and protecting workers who have experienced customer harassment by creating rest time and offering therapy and counseling. Experts said that workers’ customer service authorization systems and opinions about workplace infrastructure were also needed to implement this. Workers’ customer service authorization suggest that the manager/supervisor has the right to respond during customer harassment. In addition, it was necessary to establish systems to prepare health protection budgets for health managers and workers in the workplace [17].

Accordingly, this study identifies the latent profile types of implementation of worker protection against customer bullying and analyzes predictors that affect them. This study aims to contribute to policy development for worker protection. The specific goals of this study are as follows:

- First, to determine the general and occupational characteristics of the subjects.
- Second, to determine the latent profile types according to the implementation of protection against customer bullying.
- Third, to determine the implementation level of protection against customer bullying according to the latent profile types model.
- Fourth, to determine the predictors of the latent profile types implementing protection against customer bullying.

## Methods

### Research design

This was a descriptive study to verify the latent profile types of protection against customer bullying in workplaces and to analyze the factors affecting each type.

### Participants and data collection

The participants included customer service employees in sales, health and social welfare facilities, call centers, visiting service, and others who understood the purpose and intent of this study and agreed to participate.

Data were collected over 28 days from March 2 to 30, 2020. The researcher explained the purpose and intent of this study and obtained consent from the managers of seven organizations, such as labor unions and associations to which workers in person-to-person service belong, after which questionnaires were distributed to participants through their managers. The study’s purpose and confidentiality procedures were fully explained to the participants. To prevent the manager from seeing the responses of the employees, the questionnaire was provided in a sealed envelope that was collected when employees had completed their responses. A total of 1,550 copies of the questionnaire were collected, out of which data of 1,537 participants were included in the final analysis after excluding incomplete responses. This study was approved by the Institutional Review Board of Catholic University (MC21QISI0022).

### Research tools

#### Implementation of a protection system against customer bullying

Sub-items of the implementation of the law regarding customer bullying include preventive management, follow-up support, authorization of workers’ customer service, workplace infrastructure establishment, and welfare promotion related to customer service. Each sub-item was scored “1” for Yes and “0” for No and/or Don’t know in response to the question about whether the Occupational Safety and Health Act was implemented at their workplace; the scores were then added.

The Occupational Safety and Health Act measures for preventing health impairments caused by the abusive language of customers include: 1) posting texts or providing voice announcements to prevent abusive language, 2) creating a customer service manual including measures against customer bullying, and 3) providing education related to the contents of the customer service manual and prevention of health impairments. The items of preventive measures are rated on a scale of 0 to 3 points.

Follow-up measures for preventing health impairments caused by abusive language of customers in the Act include: 1) temporarily suspending operations and transferring workers, 2) increasing rest time, 3) offering therapy and counseling related to health impairments caused by abusive language, and 4) providing support necessary for pressing charges or claiming damages due to abusive language. The items of follow-up measures are rated on a scale of 0 to 4 points.

Workers’ customer service authorization has three items: prohibiting unfair treatment of workers who have disputes with customers; allowing workers the discretion to refuse service against immoderate demands; and implementing a control system for customers causing problems. These items are rated on a scale of 0 to 3 points.

Workplace infrastructure establishment has six items: clarifying the business policy to protect the health of workers, assigning health managers or psychological counselors to protect workers, compiling a separate budget, surveying customer bullying, establishing a grievance committee, and operating a system for workers to submit complaints or requests. These items are rated on a scale of 0 to 6 points.

Welfare promotion due to workers’ customer service has five items: offering regular counseling for customer service workers with professional counselors; running a healing program for stress relief; reinforcing working conditions such as establishing rest areas; offering pay for emotional labor; and providing a break or leave in return of emotional labor. These items are rated on a scale of 0 to 5 points.

#### General characteristics

The general characteristics of the subjects were analyzed in terms of age, gender, service period, position, and occupational category. Position was classified into staff and assistant manager or higher. Occupational category was classified into sales, health and welfare, call center, visiting service, and others.

#### Worker monitoring scope

The scope of managers’ monitoring of the workers was determined by asking about: “the manager pretending to be a customer and monitoring the workers,” “checking customer complaints online,” “installing CCTVs,” “recording conversations,” ‘the manager constantly checking the workers,” “the manager checking conversations on a computer,” and “others.” The participants were asked to check all relevant items, each of which was scored “1” and added up.

#### Changes due to protection against customer bullying

Changes due to reduced customer bullying were measured using three items: “the number of customers causing problems decreased,” “the number of disputes with customers decreased,” and “worker satisfaction increased.” These items were rated on a scale of 1 (Strongly disagree) to 5 (Strongly agree), depending on whether there was a change after implementing protective measures for customer service workers.

### Data analysis

Data were analyzed using Mplus 8.5 and SPSS 26.0, in the following order according to the research objectives.

- First, the general characteristics of the subjects were determined by obtaining the frequency, percentage, mean, and standard deviation.
- Second, latent profile types were determined according to preventative management, follow-up support, authorization of workers’ customer service, workplace infrastructure establishment, and welfare promotion related to customer service for person-to-person services. The Akaike information criterion (AIC), the Bayesian information criterion (BIC), sample size adjusted BIC (saBIC), the Lo-Mendell-Rubin (LMR) likelihood ratio test, and the bootstrap likelihood ratio test (BLRT) were employed to compare the models to determine the latent profile types.
- Third, an ANOVA was conducted to determine the implementation of worker protection measures according to the latent profile types, and ex-post analysis was conducted using the Scheffperson-
- Fourth, in order to verify the factors affecting each latent profile type, multinomial logistic regression analysis was conducted with general characteristics, worker monitoring scope, and changes after implementing protective measures.

## Results

### General characteristics

The general characteristics of the participants are shown in Table 1. Of the total participants, 32.5% were aged 40–49 years and 31.8% were aged 30–39; the average age was 40±9.7 years. Male participants constituted 36% and females 64% of the total sample size. As regards service period, 31.6% had worked for 10–19 years and 25% for 5–9 years; the average service period was 9.1±6.7 years. Of the total, 89.9% were general staff, 69.1% were in sales, and 14.4% in the visiting service.

**Table 1:** General characteristics of participants

Characteristics	Categories	N	%
Age	< 29	260	16.9
	30-39	489	31.8
	40-49	500	32.5
	50 ≤	288	18.8
	M±SD	40.0±9.7	
Gender	Male	554	36.0
	Female	983	64.0
Service period	< 1	53	3.5
	1−3	258	16.8
	3−5	225	14.6
	5−10	385	25.0
	10−20	486	31.6
	20 ≤	130	8.5
	M±SD	9.1±6.7	
Position	Staff	1381	89.9
	Assistant manager or higher	156	10.1
Occupational category	Sales and A/S	1062	69.1
	Health and social welfare	91	5.9
	Call center	65	4.2
	Visiting service	221	14.4
	Others	98	6.4

### Latent profile type of customer bullying

The latent profile type of customer bullying was explored (see Table 2). In Group 6, the LMR and BLRT were not significant; in Groups 4 and 5, they were significant. As there was less than 5% group in Group 5, Group 4 was selected as the final model. In group 4, Type 1, 2, 3, and 4 were 66.6, 14.9, 13.5, and 5%, respectively.

**Table 2:** Suitability of latent profile type according to implementation of the protection system against customer bullying

Group number	AIC	BIC	saBIC	LMR	BLRT	Latent profile type distribution rate (%)					
						1	2	3	4	5	6
1	23043.842	23097.218	23065.450	N/A	N/A	100.0					
2	19319.272	19404.673	19353.845	<.001	<.001	86.6	13.4				
3	18064.035	18181.462	18111.574	<.001	<.001	79.0	15.9	5.1			
4	17632.804	17782.256	17693.307	.012	<.001	66.6	14.9	13.5	5.0		
5	17263.450	17444.928	17336.919	.013	<.001	66.6	15.1	9.8	3.9	4.8	
6	16947.902	17161.406	17034.336	.685	1.000	66.6	16.1	5.5	3.1	4.0	4.8

*Note*: *AIC* Akaike information criterion, *BIC* Bayesian information criterion, *saBIC* sample size adjusted BIC, *LMR* Lo-Mendell likelihood ratio test, *BLRT* bootstrap likelihood ratio test

Group 6 had the lowest numbers of AIC, BIC, and saBIC. However, the LMR and BLRT were not statistically significant. In the case of Group 5, there was one type with less than 5%, so it was difficult to select an appropriate model. Therefore, Group 4 was the most suitable for the model.

**Fig. 1:**
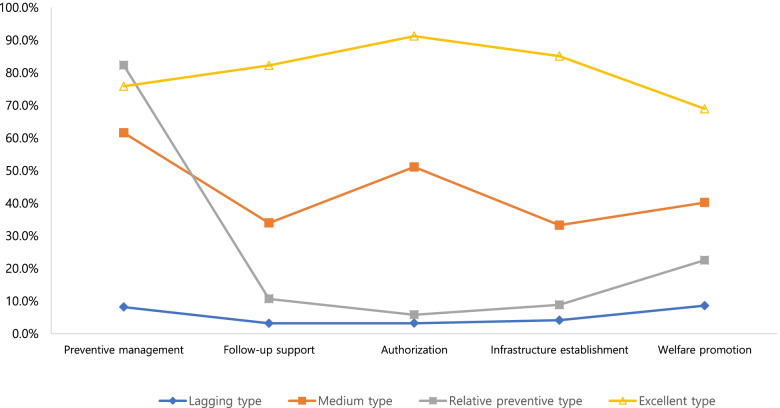
Latent profile type according to implementation of the protection system against customer bullying

Characteristics of each latent type are shown in Table 3 and Fig. 1. The mean score in Type 1 was: preventive management 0.2±0.4; follow-up support 0.1±0.4; authorization 0.1±0.3; infrastructure establishment 0.2±0.6; and welfare promotion 0.4±0.8, with all protective measures not properly implemented, and, therefore, it was named the “lagging type.” In Type 2, it was: preventive management 1.8±1.0; follow-up support 1.4±0.9; authorization 1.5±0.6; infrastructure establishment 2.0±1.4; and welfare promotion 2.0±1.2, with all protective measures implemented at a moderate level, and, therefore, it was named the “medium type.” In Type 3, it was: preventive management 2.3±1.2; follow-up support 0.4±0.6; authorization 0.2±0.4; infrastructure establishment 0.5±0.8; and welfare promotion 1.2±1.1, showing great preventive management, and, therefore, it was named the “relative preventive type.” In Type 4, it was: preventive management 2.5±0.5; follow-up support 3.3±0.8; authorization 2.7±0.5; infrastructure establishment 5.1±1.1; and welfare promotion 3.4±1.5, with most of the preventive measures greater than in other types, and, therefore, it was named the “excellent type.”

**Table 3:** Implementation of the protection system against customer bullying according to the latent profile type model

Characteristics	Lagging type (Type 1)	Medium type (Type 2)	Relative preventive type (Type 3)	Excellent type (Type 4)	F(p)
	(*N* = 1,021)	(*N* = 210)	(*N* = 230)	(*N* = 76)	
Preventive management	0.2±0.4^a^	1.8±1.0^b^	2.3±1.2^c^	2.5±0.5^d^	1161.456(<.001) a<b<c<d
Follow-up support	0.1±0.4^a^	1.4±0.9^c^	0.4±0.6^b^	3.3±0.8^d^	1017.254 (<.001) a<b<c<d
Authorization	0.1±0.3^a^	1.5±0.6^b^	0.2±0.4^a^	2.7±0.5^c^	1779.022 (<.001) a<b<c
Infrastructure establishment	0.2±0.6^a^	2.0±1.4^c^	0.5±0.8^b^	5.1±1.1^d^	1009.973 (<.001) a<b<c<d
Welfare promotion	0.4±0.8^a^	2.0±1.2^c^	1.2±1.1^b^	3.4±1.5^d^	369.186(<.001) a<b<c<d

Values with superscript letters a,b, c and d are significantly different acorss rows(*p*<.05)

The range of possible scores is different for the different indicators and the means cannot be compared between indicators, only between profiles. In Fig. 1, the Y-axis is presented as a percentage so that it can be compared between profiles.

### Factors affecting the latent profile types

All factors were used according to the derived latent profile types to conduct multinomial logistic regression analysis (see Table 4). The regression model was significant (χ2=2558.639, df=36, *p*<0.001), the Cox and Snell R2 was .251, and the Nagelkerke R2 was .292. When comparing Type 2 (medium type) with Type 1 (lagging type) as the reference group, age (OR=0.972, *p*=0.022), gender (OR=6.507, *p*<0.001), service period (OR=1.005, *p*=0.001), position (OR=2.677, *p*<0.001), call centers (OR=4.289, *p*=0.008), decrease in the number of customers causing problems (OR=0.666, *p*=0.009), decrease in disputes with customers (OR=1.379, *p*=0.047), and worker satisfaction (OR=2.049, *p*<0.001) were significant.

**Table 4:** Factors affecting latent profile types

Characteristics	Categories	Comparison group (Ref= Lagging type (Type 1))								
		Medium type (Type 2)			Relative preventive type (Type 3)			Excellent type (Type 4)		
		OR	*p*	95% CI	OR	*p*	95% CI	OR	*p*	95% CI
Age		**0.972**	**0.022**	**0.948-0.996**	0.998	0.823	0.976-1.019	0.972	0.150	0.936-1.010
Gender (ref= male)		**6.507**	**< 0.001**	**4.069-10.405**	**2.757**	**< 0.001**	**1.923-3.953**	**3.207**	**< 0.001**	**1.723-5.966**
Service period		**1.005**	**0.001**	**1.002-1.007**	1.001	0.541	0.998-1.003	1.002	0.258	0.998-1.006
Position (ref= Staff)		**2.677**	**< 0.001**	**1.592-4.501**	**2.174**	**0.003**	**1.314-3.596**	**4.680**	**< 0.001**	**2.402-9.118**
Occupational category (ref=other)	Sales	2.038	0.102	0.868-4.783	**2.219**	**0.034**	**1.060-4.645**	1.361	0.604	0.425-4.363
	Health and social welfare	1.966	0.199	0.701-5.518	1.138	0.798	0.422-3.071	2.019	0.326	0.497-8.208
	Call center	**4.289**	**0.008**	**1.466-12.542**	2.449	0.079	0.902-6.650	1.398	0.728	0.212-9.232
	Visiting service	0.403	0.108	0.134-1.219	0.580	0.230	0.238-1.413	0.201	0.090	0.031-1.285
Worker monitoring scope		1.071	0.387	0.917-1.251	0.974	0.718	0.843-1.124	**1.391**	**0.001**	**1.138-1.702**
Decrease in the number of customers causing problems		**0.666**	**0.009**	**0.491-0.903**	0.781	0.083	0.590-1.033	0.876	0.596	0.537-1.430
Decrease in disputes with customers		**1.379**	**0.047**	**1.005-1.893**	1.310	0.067	0.981-1.751	**2.177**	**0.004**	**1.275-3.718**
Worker satisfaction		**2.049**	**< 0.001**	**1.664-2.524**	**1.419**	**< 0.001**	**1.176-1.711**	**2.234**	**< 0.001**	**1.577-3.165**

-2 Log Likelihood = 2558.639, df=36, *p*= <.001

Cox and Snell R^2^ = .251, Nagelkerke *R*^2^ = .292

When comparing Type 3 (relative preventive type) with Type 1 (lagging type) as the reference group, gender (OR=2.757, *p*<0.001), position (OR=2.174, *p*=0.003), sales (OR=2.219, *p*=0.034), and worker satisfaction (OR=1.419, *p*<0.001) were significant.

When comparing Type 4 (excellent type) with Type 1 (lagging type) as the reference group, gender (OR=3.207, *p*<0.001), position (OR=4.680, *p*<0.001), worker monitoring scope (OR=1.391, *p*=0.001), decrease in disputes with customers (OR=2.177, *p*=0.004), and worker satisfaction (OR=2.234, *p*<0.001) were significant.

## Discussion

This study identified the latent profile types of protection against customer bullying in workplaces and reviewed their predictors. First, there were four types of latent profile for protection against customer bullying, namely, the lagging type (Type 1), the medium type (Type 2), the relative preventive type (Type 3), and the excellent type (Type 4). Second, their predicting variables included age, gender, service period, position, occupational category, worker monitoring scope, decrease in the number of customers causing problems, decrease in disputes with customers, and worker satisfaction. This section will discuss the characteristics of each type as well as the variables predicting these types based on the research findings.

The lagging type constituted the majority of the sample size, accounting for 66.6% of all participants. This type represented a low implementation level in all five domains of protection against customer bullying, such as preventive management, follow-up support, authorization, infrastructure establishment, and welfare promotion. This group showed the lowest possibility for the implementation of protective measures against customer bullying in the face-to-face service business of Korea and can be set as a standard for research on protection systems.

The relative preventive type, accounting for 13.5% of all participants, showed that sales and worker satisfaction are predictors. Sales is a typical occupational category of customer contact, and the demand for preventive programs against the risk of physical violence from customers has been raised for this category earlier than for other occupational categories [18]. This type includes posting texts that prevent abusive language, creating a manual to handle issues of customer bullying, and educating employees on this topic. Worker satisfaction increased merely by posting texts requesting customers to abstain from using abusive language and educating workers on how to handle abusive customers. Guay et al. [19] and Kwak et al. [20] favored education programs to teach workers the skills to handle violence, manage behaviors, and intervene in assaults. Workers must be trained to discontinue their work when customer bullying is severe and get away from the situation, and problem-solving strategies must be established to cope with customer assaults [21]. Allen [22] claimed that violence prevention programs were effective in reducing the number of assaults; however, statistically significant results for decrease in disputes with customers were not show in this study for this type. The simplest and most basic method to negate customer bullying is to make customers aware that they should not bully workers. The fact that workers’ satisfaction increases merely by knowing how to cope with bullying has significance.

The medium type, which comprised 14.9% of all participants, showed a medium-level effort to implement a worker protection system. The predictors of this type were call centers, increase in the number of customers causing problems, decrease in disputes with customers, and worker satisfaction. Issues were constantly raised in call centers because anonymous customers used abusive language. Workers are penalized if they hang up first, even if customers are using abusive language, which is why there must be a system to prevent this penalty [23]. The medium type had both the increase in the number of customers causing problems and decrease in disputes with customers, concurrently. When a worker protection system is implemented, customer complaints may increase as they may not understand workers’ actions to prevent abusive language of customers. As both customers and workers now know how to report violence against them, it increases the reporting of workplace violence [24, 25]. Previous studies show that workers tend to “sabotage” customers with problematic behavior [26, 27]. It is necessary to seek both worker protection and customer satisfaction by overcoming this vicious cycle through the mechanism of trust between customers and workers [28].

The excellent type, adding up to a mere 5% of all participants, showed high implementation rate for all five domains, namely preventive management, follow-up support, authorization, infrastructure establishment, and welfare promotion. The predictors included worker monitoring scope, decrease in disputes with customers, and worker satisfaction. In this study, worker monitoring scope refers to a system that monitors not customers but workers and whether they treat customers well. Choi et al. [29] discovered that more surveillance and monitoring by the organization had a negative impact on workers. In this study, it was a predictor of workplaces with high worker protection. Civilotti et al. [30] claimed the necessity to monitor workers for effective violence prevention programs as well as the decrease of customers causing problems. A reason for workers’ violence exposure is the lack of management attention to workplace violence, and security cameras and video monitoring must be set up to reduce violence [31]. Even though monitoring systems aim to increase customer satisfaction, they also seemed to have an effect on reducing customer violence. The monitoring system in this study showed an effect on both customer satisfaction and worker protection. This indicates that the monitoring systems in disputes with customers serve as an intervention mechanism. Given that a mediator is necessary to resolve bullying issues [32], monitoring can be used as evidence when a mediator determines whether there was bullying or not, thereby making fair judgments. This implies that the excellent type serves as the biggest predictor for the decrease in disputes with customers and worker satisfaction.

In this study, the number of men in the excellent latent type was 3.207 fold higher than that of the lagging type. In a study by Dassisti et al. [33], women were the main victims of bullying due to sexual harassment and stalking at work. When a system for managing customer harassment is implemented, women will use it more frequently than men. The government needs to provide support to protect workplaces with a high proportion of women.

Additionally, this study revealed that worker protection had an effect on decrease in disputes with customers and increase in worker satisfaction. Even with an existing law on worker protection, 66.6% of all respondents in this study reported the absence of a protection system. As service workers interact with customers the most, customer bullying is bound to have a significant impact on workplace safety of workers [22]. If there is no system for prevention or management of workplace violence, workers cannot properly handle violent situations, which will negatively impact their health [30]; therefore, worker protection systems mitigate health risks [34]. Moreover, it is necessary to establish protective systems suitable for each occupational category with the participation of managers and stakeholders such as employee representatives and health care managers [35].

In this study, the predictors were most prominent when there were several protective measures, a lot of women, and a greater number of assistant managers or executives, superior to the general staff. In a few of the types, age and service period also had a significant effect. There were more women in call centers or sales, where there is a lot of customer interaction [36], and, therefore, more women exhibited negative health effects owing to abusive language of customers [35]. Protective measures were first implemented for this occupational category, and there was a better system for senior positions and people with longer service periods. As for age, the results of this study were consistent with another study, which reported greater violence exposure for younger employees [37].

This study has a few limitations. First, it was a cross-sectional research based on the self-report of participants, and thus, cannot accurately express causal relations. Second, the participants were limited to a few occupational categories, thereby not representing all service categories. Nonetheless, this study is significant in that it identified the latent profile types of protection against customer bullying in workplaces and determined their predictors, thereby setting the policy direction.

This study makes the following suggestions. First, even though it has been a while since the bill for worker protection is passed, 66.6% of customer service employees responded that a protection system was not properly implemented. It is, therefore, necessary to inform customers to not bully the workers and educate workers on how to deal with customer bullying. Second, the medium type showed significant results on both the increase in customers causing problems and the decrease in disputes with customers. There is a need for methods to increase customer understanding of the worker protection system and promote trust between workers and customers. Third, the excellent type showed significant results for the worker monitoring system and the decrease in disputes with customers. Managers must make a balanced intervention to enhance worker competency for customer satisfaction while also reducing instances of customers causing problems, thereby satisfying both workers and customers.

## Conclusions

This study identified the implementation types of the worker protection system against customer bullying and determined their predictors. The results showed that there were four latent types of protection against customer bullying, namely the lagging type, the medium type, the relative preventive type, and the excellent type. The predictors included age, gender, service period, position, occupational category, worker monitoring scope, decrease in the number of customers causing problems, decrease in disputes with customers, and worker satisfaction.

The results indicated that preventive measures must first be implemented in the service business for protection against customer bullying. Moreover, there may be conflicts between customers and workers when protective measures are adopted, which is why trust between them must be increased. Worker protection systems against customer bullying must not conflict with customer satisfaction, but must achieve both worker protection as well as customer satisfaction simultaneously.

## Data Availability

The datasets generated and/or analyzed in this study are not publicly available but are available from the corresponding author on reasonable request.

## Abbreviations

AIC: Akaike information criterion
BIC: Bayesian information criterion
saBIC: Sample size adjusted BIC
LMR: Lo-Mendell-Rubin likelihood ratio test
BLRT: Bootstrap likelihood ratio test
